# TIDES: examining the influence of temporal individual differences on multitasking in educational simulation

**DOI:** 10.1186/s41077-020-00144-y

**Published:** 2020-11-09

**Authors:** Ashley E. Franklin, Laura Thielke, Gregory E. Gilbert, Mary Waller

**Affiliations:** 1 Harris College of Nursing and Health Sciences, Texas Christian University, Box 298620, Fort Worth, TX 76129 USA; 2 ƩɩgmaƩtats ® Consulting, LLC, 1865 Bairds Cove, Charleston, SC 29414 USA; 3 Biostatistics and Medical Writing, Real World Evidence Strategy & Analytics, ICON Commercialisation & Outcomes Services, 2100 Pennbrook Pkwy, North Wales, PA 19454 USA; 4 Texas Christian University and University of North Texas Health Science Center School of Medicine, Box 298530, Fort Worth, TX 76129 USA

**Keywords:** Multitasking, Temporal individual differences, Simulation, Balanced time perspective, Cognitive workload

## Abstract

**Background:**

The majority of tasks nurses complete in acute care settings are time-sensitive. Due to complex patient needs, nurses’ multitasking behavior is of growing importance. Situations involving multitasking behavior typically require nurses to switch their attention among multiple tasks and patients in a rapid fashion. Research suggests temporal individual differences such as time urgency, polychronicity, and time perspective influence decision-making. The factors suggest that balanced time perspective may facilitate multitasking. Given novice nurses commit errors related to multitasking, we evaluated the relationship between temporal individual differences, cognitive workload, and multitasking behaviors in a simulation setting.

**Methods:**

A one-group repeated measures design was used to evaluate the relationship between multitasking, demographic factors, cognitive workload, and temporal individual differences. One hundred sixty fourth-year, prelicensure nursing students independently completed two 45-min multiple patients simulations involving care of three interactive patient simulators. Participants completed the Multitasking Preference Inventory, Time Perspective Inventory, Experiences of Time survey, and Time Urgency Scale before simulation. A summary Creighton Simulation Evaluation Instrument score was used to represent multitasking. Participants completed the Task Load Index to represent cognitive workload. We calculated deviation from balanced time perspective and measured its correlation with multitasking. Regression models calculated how much variance deviation from balanced time perspective, demographic factors, and cognitive workload contributed to multitasking.

**Results:**

Standardized test scores were more predictive of multitasking than deviation from balanced time perspective (β = 0.19, *t* = 2.48, *p* = 0.0142). As deviation from balanced time perspective increased, multitasking behaviors decreased (*r* = − 0.17), participants reported a higher sense of urgency (*r* = 0.39), and they had more frustration after simulation (*r* = 0.22). Deviation from balanced time perspective did not influence cognitive workload.

**Conclusions:**

Nursing students who demonstrate multitasking behaviors tend to have a more balanced time perspective. Knowing students’ deviation from balanced time perspective may help educators anticipate who will need more assistance with multitasking in simulation. Nursing students frequently wait until just before graduation to provide care for multiple patients; including mention of deviation from balanced time perspective in simulation preparation may help senior nursing students become more self-aware and ultimately improve behavioral performance.

## Background

The majority of tasks nurses complete in acute care settings are time-sensitive—especially assessment and medication administration at change of shift. Nurses’ multitasking behavior and strategies to improve multitasking are of growing importance related to patients’ complex health needs [1]. Large numbers of novice nurses enter the workforce each year, and because nurses’ work is time-sensitive, it is important for novice nurses to switch their attention among multiple tasks in order to manage multiple patients’ assessments, medication administration, and procedures. Multitasking in the context of nursing care delivery represents both cognitive and psychomotor skills. It is difficult to understand why novice nurses may fail to recognize deteriorating patient conditions and how to ascertain whether they are completing the task of assessment versus interpreting assessment data [2]. Novice nurses are known to commit medication errors related to wrong dose, wrong route, and administration technique [3]. Fifty percent of all medical errors involve novice nurses with less than 1-year experience [4]. Existing research indicates the potential for significant negative effects on decision-making outcomes from stress induced by perceived time pressure [5], and time pressure perceptions are in turn influenced by temporal individual differences [6]. Given that novice nurses commit more errors related to individual tasks and to multitasking in time-sensitive patient situations [7], we chose here to evaluate the relationship between temporal individual differences and multitasking behaviors in a controlled, simulation laboratory setting.

Multitasking is inextricably linked to how individuals react to and regard time [8]. Nurses usually provide care for between four to seven patients at a time over the course of a 12-h shift [9]. Multitasking situations typically require nurses to switch their attention among multiple tasks and patients in a rapid manner [7, 10]. Nurses starting a day shift, for example, typically assess their four to seven patients, prioritize *pro re nata* medications like insulin before breakfast, and administer daily medications all during the first two hours of their shift; nurses may plan 5 min to complete each task per patient, but they are often interrupted at the start of shift with new laboratory results, orders, and interprofessional rounds. As a result, many nurses perceive inadequate time resources in which to complete their work. In situations of time scarcity, temporal individual differences—that is stable individual traits pertaining to how we perceive, consider and react to time—may become important predictors of multitasking behavior [11]. Three such differences have received considerable attention in the management and social psychology literatures over the past two decades: time urgency, polychronicity, and time perspective.

Time urgency is a temporal individual difference and subcomponent of the type A behavior pattern [12, 13] associated with time-related behaviors such as scheduling, list making, and hurrying (see Table 1). Polychronicity is an individual’s preference for doing multiple tasks simultaneously [15], whereas multitasking represents the behavioral aspect of a polychronicity preference. Finally, time perspective is a bias in the temporal frames—past, present, or future—used by an individual in tasks such as planning and decision making [16]. For example, in advising novice trainees, a mentor with a more “past time perspective” might provide substantially more examples from their own past to show how procedures and values have changed, while a mentor with a more “present time perspective” might encourage trainees to focus and reflect on their own current experiences; in contrast, a mentor with a more “future time perspective” might emphasize the importance of thinking ahead and planning next steps more often. Because of the present and future orientation of most work tasks in organizations, research on time perspective has focused on present and future orientations [17]. While being oriented to notice the passage of time (i.e., time urgent) and preferring to perform multiple tasks simultaneously (i.e., polychronic) are individual differences facilitating the switching among several tasks, a general orientation toward the future rather than present may allow individuals to better plan for multitasking; conversely, a more present orientation could lead individuals to become more single-task focused or “lost in the moment” and less able to multitask [18]. Further, individuals with a balanced time perspective (BTP)—that is, relatively equal levels of all past, present, and future time perspectives and the ability to switch among the perspectives to better fit situational demands—could be the most flexible and able to multitask at the appropriate moments [8, 19]. Table 2 depicts specific characteristics associated with temporal individual differences.

**Table 1:** Behavior pattern characteristics [14]

Type A	Type B	Type C
Ambitious Hard-working Impatient Easily aroused hostility Exaggerated sense of time and urgency	Relaxed Non-competitive Easy-going Tolerant Reflective Imaginative	Suppress emotions Pathologically nice Avoid conflict High social desirability Compliant Patient

**Table 2:** Characteristics associated with temporal individual differences [12, 15, 16, 20]

**Time urgency**	**Multitasking**	**Polychronicity**
-Time awareness -List making -Deadline control -Scheduling	-Simple tasks that require fast switching -Length of time is context-specific, but generally regarded as sixty minutes or fewer	-Prefer completing several activities at the same time -Relationship-oriented -Tolerate interruptions -Less regard for time constraints -Elaborate information networks
**Time perspective**		
**Past**	**Present**	**Future**
Recall past situations with memories of associated costs and benefits. A focus on the past affects interpretation of current decisions; top-down decision making	Focus on an individual situation, its intensity, and social aspects in order to decide on a course of action; bottom-up decision making	Anticipate and develop expectations about the future and makes decisions about present actions based on the potential cost and reward; top-down decision making
**Balanced time perspective**		
An idealized mental framework allowing individuals to switch between time perspective frames depending on situational demands, resources available, personal, and social appraisals; a compromise among the representations of past experiences, present desires, and future consequences; related to emotional intelligence		

Researchers have used time perspective to predict both psychosocial outcomes and behavior in undergraduate students from female-dominated classes similar to nursing [21]. Recent theoretical work suggests that individuals with BTP are able to attain a higher level of situational awareness than temporally skewed counterparts by switching among temporal foci as dynamic situations change—an ability hypothesized to facilitate multitasking [8]. More specifically, calculating a deviation from balanced time perspective (DBTP) as a measure of fit [20, 22] most accurately represents the nature of a balanced time perspective. A DBTP value close to zero indicates the theoretical ideal of a near balanced time perspective, where a large positive value indicates time perspective is skewed and expected to be maladaptive [20]. The shape of a DBTP curve is expected to be parabolic. In the setting of individuals planning for retirement and future behavior, those with a DBTP close to zero are able to make future plans informed by an evaluation of past experiences and current lifestyle, while individuals with a higher DBTP would consider their present lifestyle with more weight [23]. In general, having an unbalanced or skewed time perspective leaves one vulnerable when task situation demands fail to match one’s constrained time perspective preference and behaviors; conversely, having a balanced time perspective allows one to adapt time perspective and behavior to fit demands of the situation at hand.

The simulation laboratory is an ideal setting to examine the influence of temporal individual differences on multitasking behaviors. Simulation mimics the reality of a clinical environment providing novice nurses with opportunities to demonstrate critical thinking, decision-making, and behavioral procedures [24]. In multiple patients simulation, we can recognize nursing students’ preferred work speed to an extent by their low behavioral performance scores in a time-sensitive scenario^1^. There is a clear role for assessment of nursing student performance in simulation [25], and confounding variables related to temporal individual differences may help us understand why simulation-based education is effective. It is common for researchers to look for relationships between simulation performance and demographic variables including age, grade point average (GPA), and learning style preference. In meta-analyses, it is clear that simulation-based education has substantial effects on healthcare providers’ behavior change compared to no intervention [26], and simulation-based education is superior to non-simulation-based training to impact skills and behaviors [27]. Both of these meta-analyses found heterogeneity in the results; however, suggesting simulation activities vary in degrees of effectiveness. As such, simulationists seek to understand mechanisms underlying differences in simulation effectiveness (i.e., What makes simulation-based education work better? [24]). Since 2010, simulation researchers have sought to understand how to use the right type of simulation with the right learner and the right objective at the right time [28, 29]. This study seeks to understand how to use multiple patients simulation with fourth year, prelicensure nursing students and examine how temporal individual differences contribute to effective multitasking as the “right objective.”

During educational simulation, students have opportunities to practice multitasking in a realistic environment without potential danger to patients. Multiple patients simulation offers novice nurses a safe place to practice managing multiple responsibilities. Previous research indicates that multiple patients simulation increases nursing students’ motivation and self-efficacy for providing independent nursing care [30]. A gap in the literature persists however in understanding factors underpinning improved behavioral performance. As a result, we do not know whether nursing students learning how to “do simulation” over time, if facilitation skills and debriefing contribute more to improved behavioral performance than simulation practice, or if there are personality traits or other factors contributing to effective multitasking.

This descriptive study addressed the following research questions: “Is deviation from balanced time perspective more predictive of multitasking behavior in multiple patients simulation compared to demographic factors or cognitive workload in novice nurses?” and “What are the relationships between novice nurses’ deviation from balanced time perspective and their polychronicity, time urgency, flexibility, and frustration after multiple patients simulation?”

## Methods

We used a one-group repeated measures design to examine the impact of temporal individual differences on multitasking behaviors in two 45-min multiple patients simulations in which participants worked independently. This study was conducted in the simulation laboratory at a nursing school in the Southwestern United States. Researchers recruited a convenience sample of 164 fourth-year, prelicensure nursing students enrolled in a capstone clinical course. All nursing students were invited to participate, and there were no exclusion criteria. Prior to this simulation, all students completed 12 group simulations during the previous two semesters. Participation in the study had no effect on course grades. This investigation was approved by the Institutional Review Board (1806-109-1807) where the study took place and was conducted in accordance with the tenets espoused in the Declaration of Helsinki [31].

### Data collection

Participants completed surveys on a Qualtrics XM (Provo, UT, USA) platform after having provided consent. Participants self-reported demographic data, while researchers collected GPA and Assessment Technologies Institute (ATI) standardized test scores from academic records. Participants completed the Multitasking Preference Inventory [32], Time Perspective Inventory [16], Experiences of Time survey [33], and the Time Urgency Scale [34] in a 30-min session immediately following consent and on a separate day than simulation. The scales have evidence of reliability and evidence of validity with diverse samples of college students from the USA [12, 33], UK [35], and the Middle East [21]. Use of scales with multiple items and some reverse coding limits the bias of demand characteristics where participants may “second guess” and thus be motivated to fulfill (or thwart) study purposes.

After simulation and before debriefing, participants used the Qualtrics platform to complete the six-item National Aeronautics and Space Administration Task Load Index (NASA TLX) to measure cognitive workload [36]. The TLX requires participants to rate mental and physical demand alongside the amount of effort they exerted, success in their performance, and level of frustration. Participants self-rate on a scale of 1–20. Researchers used item-level scores for analysis of frustration and a global summary score to represent cognitive workload. The TLX also has evidence of reliability and evidence of validity [37]. Table 3 provides a summary of the constructs assessed with each measure.

**Table 3:** Summary of measures

Measure	Number of items	Timing relative to scenario	Construct	Level of analysis
Sociodemographic Questionnaire	10	Participant completes after consent, but on separate occasion than scenario	Demographic variables	Individual items
Multitasking Preference Inventory	14		Polychronicity	Global summary scores
Time Perspective Inventory	56		Time perspective	Derivative calculation of Deviance from Balanced Time Perspective^a^
Experiences of Time Survey	53		-Punctuality -Flexibility -How you talk about time	Subscale summary scores
Time Urgency Scale	33		Time urgency	Global summary score
Creighton Simulation Evaluation Instrument	57	Educator completes during scenario	Multitasking	Global summary score
NASA Task Load Index	6	Participant completes after scenario, but before debriefing	Cognitive workload	Global summary score, and frustration individual item score

^a^We used a derivate calculation for deviance from balanced time perspective (DBTP) to represent the nature of balanced time perspective more accurately [20]

### Multiple patients scenario and scoring performance behaviors

A complete description of the multiple patients scenario is available in a previous publication [38]. Briefly, the scenario involved care of three interactive patient simulators for 45-min at the beginning of shift. Educators provided a scripted orientation to the environment, and had participants had 10 min to review the electronic health record independently after receiving report. One patient required rescue medications including *pro re nata* medications for chest pain. Participants completed the scenario independently, and there were no observers. Table 4 provides details about the multiple patients scenario. Given the inherent time constraints of the beginning of shift, participants were required to establish and reorganize priorities. Debriefing was not included in data collection or analysis, though educators used a standard script with open-ended prompts (see Additional file 1 Appendix) and stated behavioral observations guided by the Creighton Simulation Evaluation Instrument™ (CSEI) using the Advocacy/Inquiry approach. The majority of participants completed both simulations before starting their capstone shifts which involved total patient care.

**Table 4:** Key elements of multiple patients scenario

Element	Subelements	Description
Participant orientation	Orientation to simulator	Participants were familiar with simulator after having completed 12 group simulations in the previous two semesters
	Orientation to environment	Educator provided 5-min scripted orientation to environment including review of operating phone system, patient monitor, Pyxis medication dispense system, and medication refrigerator for insulin. Participants had 10 min to review electronic health record independently.
Simulator type	Make and model	Three Gaumard interactive patient simulators similar to Hal® S3201
	Functionality	Pulse sites, heart sounds, ECG rhythms, lung sounds, chest rise, wireless streaming audio, eye blinking, touchscreen vital signs monitor, bowel sounds
Simulation environment	Location	Simulation center with one double patient room and one single patient room used by each participant, central control room, and separate medication room
	Equipment	Electronic health record, medication room with supplies for participants to choose from, Pyxis medication dispense system, simulation phone system, AV system with cameras capturing all spaces for raters to view via live stream
	External stimuli	In the large simulation center, one participant completed scenario at a time using two patient rooms and a medication room. Participants interacted with an embedded participant in the role of nursing assistant. Participants gathered their own supplies from the medication room as needed.
Simulation scenario	Event description	Scripted orientation, prebrief, scenario progression, and triggers. One patient experienced chest pain at 30-min mark. Scripts available in appendix.
	Learning objectives	1. Prioritize which patient to see first 2. Individualize safety checks and focused assessments 3. Communicate therapeutically 4. Administer medications safely Educator provided orientation to objectives during scripted orientation to environment. Participants reported which patient they wanted to see first in order to help educator know how scenario would begin.
	Group vs. individual practice	Individual practice. There were no observers. Each participant completed two scenarios
	Use of adjuncts	Moulage for wounds and dressings. Alaris IV pumps running during scenario. Participants administered medications for IV and subcutaneous routes. Headwall allowed for O_2_ titration.
	Operator characteristics	Two nurses with 2 years’ experience as simulation technician operated the simulator and provided scripted cues
	Pilot testing	> 200 individual participants in previous teaching and research over 5+ years
	Standardized patients	Two female embedded participants who work routinely in simulation center served as nursing assistant to whom participants could delegate care; one with clinical experience as medication aide; training by PI; scripted cues and vital signs provided; wore ear bud walkie-talkie for cue to notify participant of chest pain at 35-min mark
Simulation exposure	Duration	45 minute scenario representing the beginning of shift in acute care setting
	Timing	No just-in-time training or educational intervention before scenario
	Frequency	Two multiple patients scenarios completed approximately four weeks apart
	Clinical variations	All participants provided care for the same patients. Patients had diagnoses of respiratory distress, cardiac disease, and diabetes complications. Research team mapped complexity of patients for scenario 1 and scenario 2 to ensure equivalence.
	Assessment	Rater observer measurement of behavior performance using Creighton Simulation Evaluation Instrument™. Standards established through two rater training videos and discussion to establish consensus on behavioral descriptors. Eight educators served as raters.
	Range of difficulty	Simulation patients had low acuity similar to acute medical-surgical hospitalized patient. Difficulty in scenario presented mostly on time management and priority setting with care of three patients simultaneously
	Nonsimulation interventions and adjuncts	Participants reviewed simulation preparation materials related to performing safety checks and focused physical assessments, prioritizing patient care, communication to providers using SBAR framework over the telephone, delegating to unlicensed assistive personnel, administering medications safely, and using a nurse brain tool to organize care. Preparation materials were available on learning management system in several formats (expert modeling videos, voice over PowerPoint, and reading materials) for participants to choose from according to learning style preference. Simulation preparation occurred before participants reported to the simulation center.
	Integration	Participants previously completed two group simulation courses involving 12 class days and 36 patients over the course of two academic semesters. This simulation activity represents the capstone simulation experience in a five semester prelicensure nursing curriculum. Participants completed this simulation immediately before their capstone clinical experience (internship)
Feedback	Source	Educator
	Duration	45 min
	Facilitator presence	One educator face-to-face
	Facilitator characteristics	Nurse educators with 3–30 years’ experience teaching in clinical and simulation settings. PI provided open-ended prompts and trained educators on stating behavioral observations guided by the Creighton Simulation Evaluation Instrument™ (CSEI) using Advocacy/Inquiry approach
	Content	After simulation 1, attention to focused assessment and safety domains from Creighton Simulation Evaluation Instrument™ After simulation 2, attention to communication and priority setting domains
	Structure	Reactions, Gather, Analyze, Summary format
	Timing	Debriefing immediately after simulation and after participants completed NASA TLX
	Video	After debriefing, participants watched their 45 minute simulation video and wrote a reflection about integrating suggested behaviors into their performance
	Scripting	Scripted open-ended prompts available in appendix.

Performance behaviors were scored using the CSEI [39]. The instrument has evidence of validity, reliability, and interrater reliability [40]. Scores are calculated by summing the responses, and higher scores on the CSEI represent effective multitasking. Eight educators rated participants’ multiple patients simulation, and a research assistant verified interrater reliability in a random ten percent of the sample. Gwet’s AC_2_ calculation for inter-rater reliability was used.

### Deviation from balanced time perspective

Five time perspective factors are measured with the 56-item Zimbardo Time Perspective Inventory [16]. Table 5 depicts sample items from each of the five time perspective factors. DBTP allows researchers to categorize participants individually based on their distance from a balanced time perspective profile [22]. Balanced time perspectives optimize how individuals meet situational demands of life and how they think, and how they behave. We used a formula to calculate DBTP using subscales of the Zimbardo Time Perspective Inventory:

$$ DBTP=\sqrt{{\left( oPN- ePN\right)}^2+{\left( oPP- ePP\right)}^2+{\left( oPF- ePF\right)}^2+{\left( oPH- ePH\right)}^2+{\left( oF- eF\right)}^2} $$

**Table 5:** Factors and sample questions on Zimbardo Time Perspective Inventory [16, 20]

Subscale	Sample item	Known correlations
Past positive perspective	“On balance, there is much more good to recall than bad in my past”	Warm attitude toward the past
Past negative perspective	“I often think of what I should have done differently in my life”	Depression, unhappiness, low self-esteem, anxiety
Present hedonistic perspective	“I believe that getting together with one’s friend to party is one of life’s important pleasures”	Novelty-seeking, sensations seeking, and ego under-control
Present fatalistic perspective	“Fate determines much in my life”	“Future is predestined and uninfluenced by individual action”
Future perspective	“I believe a person’s day should be planned ahead each morning”	Conscientiousness, consideration of future consequences, number of hours spent studying per week

where oPN is the optimal past negative subscale,

ePN is the empirical past negative subscale,

oPP is the optimal past positive subscale,

ePP is the empirical past positive subscale,

oPF is the optimal present fatalism subscale,

ePF is the empirical present fatalism subscale,

oPH is the optimal present hedonism subscale,

ePH is the empirical present hedonism subscale,

oF is the optimal futurism subscale, and

eF is the empirical futurism subscale [20]

After calculating the deviation from balanced time perspective, it was operationalized in this investigation as a categorical variable based on tertiles (0 to 1.518030, > 1.518030 to 2.038265; and > 2.038265).

## Analysis

Descriptive statistics were calculated—means (standard deviations; SD) for quantitative data and frequencies and percentages (relative frequencies) for qualitative data. Dependent variables were tested for normality using normal probability plots and the Anderson-Darling, Shapiro-Francia, and the Shapiro-Wilk normality tests. Pearson’s product-moment correlation coefficient was used to assess the bivariate relationships [41].

Analysis of variance was used to determine if DBTP was predictive of CSEI scores. The preferred method, the Tukey-Kramer method, was used to control the comparisonwise error rate for post hoc pairwise comparisons. The paired *t* test or the Wilcoxon Signed-rank test (based on the results of normality testing) was used to assess if the first CSEI score was an important predictor of the second CSEI score. After which, analysis of covariance was used to assess of DBTP has any effect on second CSEI scores after adjusting for initial score. Linear regression was used to measure the effects of GPA, ATI, DBTP, and cognitive workload as measured by the NASA TLX. In regression models, DBTP was entered as a quantitative variable.

The family of kappa measures for inter-rater reliability have documented problems [42–44]. To resolve these paradoxes, Gwet proposed a family of inter-rater reliability statistics; the AC statistics [45]. Gwet’s AC_1_ is a measure of inter-rater reliability for nominal data for multiple raters while Gwet’s AC_2_ is a measure of inter-rater reliability for ordinal and interval measurement [46]. Gwet’s AC_2_ calculation for inter-rater reliability was used. All analyses were done using software for statistical computing (R v.3.5.2).

## Results

Table 6 depicts demographic characteristics of the sample comparing participants in three groups based on their calculated DBTP. Four participants had missing data related to surveys completed after simulation and were not included in analysis; 160 participants completed the study protocol. Data were judged to be normally distributed except for a difference between first CSEI score and second CSEI simulation score; therefore, the second CSEI simulation score was rank transformed for analysis. Inter-rater reliability (Gwet’s AC_2_) for the 10% random sample of CSEI was .78 (95% CI .76–.80).

**Table 6:** Demographics of DBTP groups

Variable	Low DBTP *n* = 54 % (*n*)	Medium DBTP *n* = 52 % (*n*)	High DBTP *n* = 58 % (*n*)
Gender			
Female	96.3 (52)	90.4 (47)	91.3 (53)
Male	3.7 (2)	7.7 (4)	8.6 (5)
Prefer not to disclose	–	1.9 (1)	–
Race			
American Indian	–	1.9 (1)	–
Asian	1.9 (1)	1.9 (1)	6.9 (4)
Black or African American	1.9 (1)	3.8 (2)	5. 2(3)
Prefer not to disclose	1.9 (1)	–	5.2 (3)
White	94.4 (51)	92.3 (48)	82.8 (48)
First-time degree seekers	77. 8 (42)	80.8 (42)	77.6 (45)
Age			
18–21 years	29.6 (16)	36.5 (19)	29.3 (17)
22–26 years	59.3 (32)	55.7 (29)	60.3 (35)
27–32 years	7.4 (4)	7.7 (4)	5.2 (3)
33–38 years	1.9 (1)	–	5.2 (3)
39–44 years	–	–	–
> 45 years	1.9 (1)	–	–
Cumulative GPA mean (SD)	3.4 (.31)	3.4 (.28)	3.3 (.28)
ATI score to predict passing National Council Licensure Examination (out of 100%; Mean (SD))	66.7 (7.9)	66.2 (8.1)	65.5 (7.8)

### Research question 1

As DBTP increased, participants’ CSEI scores decreased in the first simulation (*r* = − 0.17). In regression models adjusting for GPA, ATI, DBTP, and second NASA TLX score, results were similar. On the first multiple patients simulation, only ATI score predicted CSEI scores in a meaningful^2^ way. Approximately every one-point change in ATI score will be associated with a five-point change in the CSEI score (β = 0.19, *t* = 2.48, *p* = 0.0142). A model including GPA, ATI, DBTP, and NASA TLX score explained about 14% of the variance on CSEI score in the initial multiple patients simulation.

On the second multiple patients simulation, ATI score and DBTP predicted simulation performance scores on the CSEI. Approximately every one-point change in ATI score will be associated with a five-point change in the CSEI score (β = 0.18, *t* = 2.64, *p* = 0.009). For every five-point increase in DBTP, there was a corresponding six-point decrease in CSEI score (β = − 1.23, *t* = − 1.91, *p* = 0.059). A model including GPA, ATI, DBTP, and score on the NASA TLX explained 13% of the variance on CSEI score in the second multiple patients simulation.

Regardless of DBTP group assignment (low, medium, high), there was no meaningful difference in performance on the second multiple patients simulation performance (where participants were familiar with the environment and objectives; *F*(2, 157) = 1.703, *p* = 0.186). A Wilcoxon signed rank test demonstrated a meaningful difference between multitasking on the first and second simulations (*p* < 0.0001, *r* = 0.09). However, in an analysis of covariance of DBTP adjusting for first CSEI score, DBTP was not a predictor of the second CSEI score (*p* = 0.400). Adjusting for DBTP category did not modify the results of the model, initial CSEI score was still predictive of second CSEI score at the same level (*p* < 0.001). However, a model of DBTP and first CSEI score accounted for about 28% of the variance in the second CSEI score.

DBTP was not predictive of CSEI score. Further, DBTP did not substantially influence CSEI score or cognitive workload. However, in both simulations, ATI score was an important or meaningful predictor of CSEI score with every one-point change accounting for a five to six-point change in CSEI score. Both adjusted models accounted for minimal variance suggesting there are additional variables at play.

### Research question 2

Participants with a preference for multitasking on the Multitasking Preference Inventory [32] also reported not having urgency in responding to everyday situations (*r* = 0.25). As DBTP increased, participants reported a higher sense of urgency on the Time Urgency Scale [12] and frustration after their first multiple patients simulation (see Table 7). Physiological correlations (e.g., amount of nicotine administered to the heart and heart rate) of this magnitude may be considered low; however, behavioral indications correlations (e.g., multitasking and temporal demand) of this magnitude can be considered moderate. There were minimal relationships detected between multitasking and cognitive workload at simulation 1 (*r* = 0.14) and simulation 2 (*r* = 0.11). Regardless of DBTP group assignment (low, medium, high), there was no difference in cognitive workload after the second simulation (*F*(2, 155) = 0.6963, *p* = 0.5).

**Table 7:** Temporal individual difference correlations

	1	2	3	4	5	6
1. Multitasking	–					
2. Urgency	− .06	–				
3. Flexible thinking	− .03	.31	–			
4. Punctual	− .25	.27	.26	–		
5. Balanced time perspective (DBTP)	− .01	.39	.16	.2	–	
6. Frustration after multiple patients simulation	− .05	.29	.05	.04	.22	–
7. Pressured by time/temporal demand	− .04	.15	− .01	− .12	− .05	.34

## Discussion

Nurse educators use simulation for teaching and assessment to mitigate the gap between academics and independent nursing practice. For years, employers have provided academic nurse educators with feedback that novice nurses are ill-prepared for practice and cannot multitask or manage their time appropriately [48, 49]. This is the first study to investigate how nursing students multitask in multiple patients simulation and to examine how temporal individual differences, demographic factors, and cognitive workload relate to multitasking behaviors. Findings from this study of 160 novice nursing students indicate that DBTP was not predictive of multitasking and ATI standardized test scores were actually more predictive. DBTP did not influence cognitive workload. Regression models accounted for minimal variance suggesting variables other than DBTP, demographic factors, and cognitive workload influence novice nurses’ multitasking.

This study builds on the existing multiple patients simulation body of knowledge by identifying predictors of multitasking. Seven research teams have previously published multiple patients studies where simulation was a platform to evaluate multitasking behavior, though this study was the first to examine the relationship between multitasking behaviors, demographic factors, and nursing students’ temporal individual differences. In earlier trials, novice nurses improved in competent behavioral performance with additional practice in multiple patients simulations [38, 50, 51]. Repeated multiple patients simulation opportunities improved nursing students’ competent behavioral performance, though manipulating simulation preparation activities did not further enhance behavioral performance [52]. Findings from this study are similar as novice nurses’ multitasking behavior improves with repeated exposure in simulation; this study was unique because educators facilitated debriefing after simulation (see Additional file 1 Appendix), and that may have contributed to the meaningful difference in multitasking between simulations 1 and 2. Because hearing an expert’s observations and practicing components of a specific skill repeatedly improve behavior, educators in academic and practice settings continue to advance the platform of simulation-based education to improve healthcare providers’ performance through promoting skill acquisition, uncovering clinical variations, maintaining competence, and building efficiency in care delivery processes [25].

One of the largest challenges for nursing students to overcome is learning to manage multiple responsibilities simultaneously and independently [53]. In order to preserve patient safety, nursing students usually provide care to only one patient at a time during clinical courses. Providing care to one patient at a time helps new nurses think deeply about their patient’s condition, medications, and treatments. Although this deep, critical thinking helps student nurses develop core thinking skills, one of the downsides to only providing care to one patient at a time is it does not motivate completion of multiple tasks simultaneously. However, completing multiple tasks simultaneously is a characteristic of real-world independent nursing practice, especially because nurses provide care to four to seven patients simultaneously in the majority of acute care settings and multitask 34% of their shift (range 23–41% [54]).

We anticipated that nursing students with a balanced time perspective would multitask more effectively. Existing literature from psychology and organizational behavior indicates substantial influence of temporal individual differences on work behaviors, ranging from differences in individuals’ preferred work speeds [55], to planning for the future [56], and suggests polychronicity [57] and time urgency [58] are correlated with multitasking behavior. Although past time perspective is hypothesized to be associated with a monochronic preference [18], and both present and future time perspectives are theorized to influence reactions to working under deadlines [17], we are aware of no empirical work examining the relationship between time perspective and actual multitasking behavior in a nursing context.

Even though multitasking behaviors increased over time, our participants’ scores related to priority setting and assessment are still poor. Namely, participants earned only 29% of priority setting points and only 50% of assessment points on their second multiple patients simulation. These results are actually much worse than those reported by Kavanaugh and Szweda with a much larger sample of new graduate novice nurses using a computerized assessment of priority setting skills [53]. It could be our experiential multiple patients simulations where nursing students physically perform assessments, give medications, and respond to interactive patient simulators in a realistic setting may uncover more discrepancies related to priority setting and assessment than computerized simulation exercises. Similarly, it seems GPA may represent intellectual ability but not relate to multitasking behavioral performance. Extant literature also highlights the role of novice nurses’ values and how a preference for listening to patients and providing them with explanations about the care plan may be more important to novice nurses with 6 months of practice experience compared to nursing students in the present study [48]. Our research team observed that nursing students tended to favor completing tasks in a linear fashion (e.g., repeating the same assessment on several patients without individualizing the care plan to patient history) and to value interventions in lieu of prioritizing care. However, 75% of the points on the CSEI in the priority setting section required patient teaching about priorities of care and explanations of rationale for individualized patient goals and interventions.

Data from the NASA TLX revealed a weak correlation between level of frustration and a sense of urgency and DBTP. Though a level of frustration seems like a negative affective outcome following simulation, previous research validates how novice nurses vacillate between feeling frustrated and confident even within the confines of a single 12-h shift [48]. To our research team, levels of students’ frustration were appropriate in the setting of time scarcity and multitasking. Previous qualitative research with new graduate nurses referred to a continuum of frustration to confidence [48]; because confidence after simulation routinely does not match competent behavioral performance [59], frustration seems to be an acceptable representation of challenge and reaction to complex educational simulations. Further, because our participants completed the NASA TLX before debriefing, it is important to recognize how debriefing after difficult clinical situations naturally deescalates frustration as students reflect both on judgments underpinning behavior as well as opportunities for improvement [60].

Our findings are similar to published research about new graduate nurses and multitasking. Reflecting on their transition to practice as new graduate nurses, it is common for novices to report apprehension about completing all of their tasks and making a mistake contributes to the struggle they experience with time management in the early months of independent practice [61]. Further, novice nurses describe a tension between “being able to do time management and patient safety” [61] potentially explaining the frustration our participants experienced in a time-sensitive multiple patients simulation. Interestingly, however, this tension around multitasking seems to resolve for nurses who have practiced independently for a while. For example, researchers found that nurses view multitasking as a prerequisite for providing care in an Emergency Department because multitasking implies efficiency [62]. Further, expert nurses have identified the relationship between multitasking and patient error is perceived to be mediated by who the nurse is and their level of experience [62]. As such, it is prudent to consider lack of experience for new graduate nurses and our nursing students contributes to frustration in multiple patients simulation.

Our findings related to DBTP and multitasking are not to be considered an impasse as a research outcome. It could be one reason we did not find DBTP as predictive of multitasking relates to measurement and use of dichotomous checklists to capture multitasking behavior. Though the CSEI is widely used in simulation research and works well because nurse educators can individualize behavioral descriptors to their scenario and objectives [39, 40], the dichotomous nature of the tool may limit conclusions about multitasking behavior. More specifically, the CSEI does not account for frequency or timing of behaviors in the manner than more fluid time motion studies potentially represent [7, 10]. Further, our research team noted lack of weighting on CSEI items limits conclusions about priority setting. For example, the CSEI awards equal points for foundational skills and priority setting decisions even though the two represent varied levels of complexity. From a research perspective, it would be helpful to ascertain whether or not nursing students multitask effectively while also demonstrating higher-order priority setting skills. It is worthwhile to consider that most associations between DBTP and cognitive functions are relatively small [63], so refining the measure of multitasking in simulation and re-examining the relationship in future study is warranted.

By examining the influence of temporal individual differences in the realistic and consequential setting of the nursing simulation laboratory, we have started a conversation about predictors of effective multitasking for nursing students. Previous research has shown that “future time perspective” mediates the relationship between negative awareness of age-related change and psychological well-being in adults; the more expansive the “future time perspective,” the more healthy the sense of well-being [64]. Similarly, if a nursing student knows their DBTP is high, they might choose to gain more multitasking practice, focus more intently on task switching skill development, or seek a work environment requiring less time-pressured multitasking behavior. Simulation facilitators may find it helpful to use more Advocacy/Inquiry in debriefing with students who have a high DBTP to facilitate a higher level of self-reflection. Nurse educators can use DBTP to screen nursing students and novice nurses and in turn help nurses become more aware of their own strengths and limitations related to BTP so they are able to work independently and have a sense of well-being. The ultimate goal is to create targeted interventions helping nursing students overcome individual challenges with multitasking in order to decrease error and improve patient safety. Considering data on self-reported perceptions of temporal difference alongside rater observation of multitasking behavior, results seems promising. The next steps in this line of research are to add behavioral process into evaluation to uncover how temporal individual differences influence performance outcomes. Incorporating time motion studies to code simulation videos for the presence and frequency of behaviors should add to our understanding of factors contributing to multitasking in simulation.

This study has several educational implications. Nurse educators can use Zimbardo’s Time Perspective Inventory [16] and calculate DBTP [22] as a screening tool to understand nursing students’ time perspective balance. Information about time perspective balance combined with data on academic performance, computerized assessments of higher-order thinking skills, and simulation can help educators create an individualized education plan for at-risk nursing students and novice nurses. Educators in practice settings already use simulation to improve novice nurses’ management, priority setting, and assessment skills [48, 65]. Adding a survey of temporal individual differences may assist educators to recognize how stable traits impact nursing care and priority setting.

Nursing students preferring to work more slowly seem to revert to a comfort zone of technical skills (e.g., patient identification and vital signs) rather than taking initiative to prioritize assessment, decide what data requires the most urgent response, and follow through to deliver appropriate patient care. These students seem paralyzed or “lost in the moment” by the daunting list of tasks when managing multiple responsibilities. As a result, novice nurses, generally, are less able to assess a clinical situation or ask pertinent assessment questions enabling them to recognize a change in patient condition [66]. In planning multiple patients simulations to foster multitasking, educators should design scenarios rewarding nursing students’ assessment and priority setting in lieu of interventions in order to reinforce the unique position of nurses to recognize cues of a change in patient status and alter care plans accordingly. Further, educators should encourage nursing students to communicate their priorities to their patients in these complex simulations and draw connections between communicating, involving a patient in their care, and promoting patient safety. Finally, we implore educators to measure behavior performance with other outcome variables to further investigate predictors of effective multitasking in multiple patients simulation.

### Limitations

Sample characteristics represent a limitation to generalizability. We used convenience sampling from one university in one region of the country. As such, the sample may underrepresent some groups based on age, gender, race, ethnicity, and work experience. Findings from this study could be different if students from other universities were included in data collection. Despite this limitation, conclusions about temporal individual differences and how they impact multitasking behavior are novel to the nursing education literature.

Another limitation of this descriptive study relates to measurement of priority setting within the limited confines of a single scenario. Compared to computerized exercises capturing nurses’ priority setting and other higher-order thinking skills [53], conclusions about priority setting in this study are vulnerable to error. Results could differ if participants completed more multiple patients simulations with different populations over time.

## Conclusion

This study of the relationships between temporal individual differences, demographic factors, cognitive workload, and multitasking adds to the literature because it describes predictors of nursing student success in multiple patients simulations. Standardized test scores appear to predict effective multitasking more than temporal individual differences such as DBTP. Nursing students who demonstrate multitasking behaviors tend to have a more balanced time perspective. Those feeling time pressure in multiple patients simulation may report feeling frustrated because they were not able to complete all of the tasks they envisioned, but evidence from the practice literature confirms our findings novice nurses feel time pressure during their first months of independent practice. Knowing students’ DBTP may help educators anticipate who will need more assistance with multitasking in simulation. Nursing students frequently wait until just before graduation to provide care for multiple patients; including mention of deviation from balanced time perspective in simulation preparation may help senior nursing students become more self-aware about and ultimately improve behavioral performance.

### Supplementary information

**Additional file 1.** Appendix

## Data Availability

The dataset used and analyzed during the current study is available from the corresponding author on reasonable request.

## Abbreviations

ATI: Assessment Technologies Institute
BTP: Balanced time perspective
CSEI: Creighton Simulation Evaluation Instrument
DBTP: Deviation from balanced time perspective
GPA: Grade point average
NASA: National Aeronautics and Space Administration
SD: Standard deviation
TLX: Task Load Index

## Footnotes

1 It is difficult to parse out whether students’ behavior in simulation represents their true self or what they believe educators want to see/what educators are assessing. The bias of demand characteristics is a risk in all educational simulations and those meant for evaluation. Despite this potential confounder, simulation remains a more appropriate setting to assess nursing students’ multitasking than traditional clinical settings, especially because hospital-based educators repeatedly provide feedback that novice nurses’ problems with multitasking impact patient outcomes. Our descriptive design, without testable hypotheses, decreases the threat of demand bias interfering with findings. Participants and raters were not aware of the “answer” we wanted fulfilled in order to further protect against the bias of demand characteristics.

2 The term meaningful was used based on the *P* value. Pre-2019, we would have said ATI scores were a “significant” predictor of CSEI scores. However, in April 2019, the American Statistical Association suggested formally the term “statistical significance” be abandoned along with the terms “statistically significant,” “*p* < 0.05,” and “nonsignificant.” Wasserstein and colleagues continue imploring researchers to remove these terms and similar terminology in favor of reporting exact *p* values and interpreting difference from a practical perspective [47].
