# Correction: TIDES: examining the influence of temporal individual differences on multitasking in educational simulation

**DOI:** 10.1186/s41077-023-00271-2

**Published:** 2023-12-20

**Authors:** Ashley E. Franklin, Laura Thielke, Gregory E. Gilbert, Mary Waller

**Affiliations:** 1 Harris College of Nursing and Health Sciences, Texas Christian University, Box 298620, Fort Worth, TX 76129 USA; 2 ƩɩgmaƩtats ® Consulting, LLC, 1865 Bairds Cove, Charleston, SC 29414 USA; 3 Texas Christian University and University of North Texas Health Science Center School of Medicine, Box 298530, Fort Worth, TX 76129 USA

**Correction: Adv Simul 5, 31 (2020) **

**https://doi.org/10.1186/s41077-020-00144-y**

Affiliation #3 in the original article [1] is erroneous and should be disregarded. The correct affiliations can be viewed in this Correction article.
