# Supplementary material for: TIDES: examining the influence of temporal individual differences on multitasking in educational simulation
Source: Adv Simul (Lond). 2020 Nov 9;5:31. doi: 10.1186/s41077-020-00144-y (PMC7650193; doi:10.1186/s41077-020-00144-y)
Supplement: Supplementary file 1 — Additional file 1. Appendix [file 41077_2020_144_MOESM1_ESM.docx]

Appendix

Open-ended Debriefing Prompts

Reactions

1. How was multiple patients simulation for you?

Safety Checks

1. How did safety checks go for these patients? What came naturally? What items were difficult to remember?

Focused Physical Assessments and Priorities

1. Which patient was your top priority going in? Why?
2. What were your physical assessment findings for that patient? Were they what you expected based on report and/or pathophysiology?

Medication Administration

1. How did you prioritize who received medication first?
2. What did you notice about safety related to medication administration? What parts of medication were challenging today?

Communication and Delegation

1. What concerns did your patients have? How did you respond?
2. How did SBAR go?
3. What did you learn about delegation? Was it difficult to remember to follow-up with the tech and “close the loop?”

Miscellaneous

1. How did you handle time management during the scenario?
2. What role did patient privacy and confidentiality play in your sim? In your delegation and follow up to the nursing assistant?
3. What are your big take aways for moving forward?

**After you give the student a chance to respond to debriefing prompts, state your observations about behaviors and what you noticed was missing using the Creighton Simulation Evaluation Instrument as a guide.**

Focus on simple concepts at the beginning of the semester and more complex concepts at the end. I recommend you state the observations according to

| Focused Physical Assessments  Safety Checks | Emphasize more for simulation 1 |
| --- | --- |
| Communication  Critical Thinking and Priority Setting | Emphasize more for simulation 2 |

Here’s a script you might use to frame the conversation when you share behavioral observations:

1. The purpose of this learning activity, and the reason we are doing multiple patients simulation twice, is to help you improve your nursing care. It doesn’t matter what score you receive; rather, we are looking for improvement.
2. This semester you have opportunities to focus on your independent nursing care. I know this is different than in previous semesters when you have had clinical experiences with a group.
3. I want to encourage you to take personal accountability for your own skills and performance.
4. Today, you scored ______ points.
5. We expect your score will improve on your next simulation.
6. You can use the review materials on TCU Online related to __________ concepts (see below) to improve your performance.
7. Specifically, in _________ concept/category, I thought you did a good job at ____________,
8. Incorporate Advocacy/Inquiry approach:

-There was room to improve related to __________ (state observation).

-This area is particularly important when it comes to managing multiple patients because ________ (explain your concern).

-When you were ______, tell me what you were thinking about_______ (explore cognitive frame).

-It’s important to me that you know _____ and that when you ______ your behaviors reflect the principles of best practice. I would suggest you ______ (close the performance gap).

1. This evaluation tool is designed to capture behaviors related to assessment, safety checks, communication, and critical thinking.
2. Remember, there are resources for you online to help you prepare. What is your learning style preference?

**Refer them to simulation preparation videos online for examples of expected behaviors for**

**Taking report using a Nurse Brain tool**

**Prioritizing which patient to see first**

**Delegation to a CNA**

**Safety Checks at the Bedside**

**Focused Physical Assessments**

**Using SBAR on the telephone**

**Safe Medication Administration**
